# Lung Lipidomic Alterations in Beagle Dogs Infected with *Toxocara canis*

**DOI:** 10.3390/ani12223080

**Published:** 2022-11-09

**Authors:** Hao-Yu Li, Yang Zou, Yue Xu, Lang Cai, Shi-Chen Xie, Xing-Quan Zhu, Wen-Bin Zheng

**Affiliations:** 1 Laboratory of Parasitic Diseases, College of Veterinary Medicine, Shanxi Agricultural University, Jinzhong 030801, China; 2 State Key Laboratory of Veterinary Etiological Biology, Key Laboratory of Veterinary Parasitology of Gansu Province, Lanzhou Veterinary Research Institute, Chinese Academy of Agricultural Sciences, Lanzhou 730046, China; 3 Key Laboratory of Veterinary Public Health of Higher Education of Yunnan Province, College of Veterinary Medicine, Yunnan Agricultural University, Kunming 650201, China

**Keywords:** *Toxocara canis*, toxocariasis, Beagle dog, lung, lipidomics, lipid

## Abstract

**Simple Summary:**

Toxocariasis is mainly caused by *Toxocara canis*, and to a lesser extent, *Toxocara cati*, and is a globally distributed zoonotic parasitic disease. Lipidomics is a discipline that has been developed after genomics and proteomics, and it is an important means of studying systems biology. In this study, we used liquid chromatography–tandem mass spectrometry (LC-MS/MS) to comprehensively examine the lipidomic alterations in the lungs of Beagle dogs infected with *T. canis*, and to analyze the differential lipids to reveal their potential biological functions. There were 63, 88, and 157 lipid species that changed significantly at 24 hpi, 96 hpi, and 36 dpi, respectively. Further analysis of the functions of these differential lipids, such as triglyceride (TG), lysophosphatidylserine (LPS), and ceramides (Cer), will better reveal the mechanism of *T. canis* pathogenesis.

**Abstract:**

Toxocariasis, mainly caused by *Toxocara canis*, and to a lesser extent, *Toxocara cati*, is a neglected parasitic zoonosis. The mechanisms that underlie the changes in lipid metabolism of *T. canis* infection in Beagle dogs’ lungs remain unclear. Lipidomics is a rapidly emerging approach that enables the global profiling of lipid composition by mass spectrometry. In this study, we performed a non-targeted lipidomic analysis of the lungs of Beagle dogs infected with the roundworm *T. canis* using liquid chromatography–tandem mass spectrometry (LC-MS/MS). A total of 1197 lipid species were identified, of which 63, 88, and 157 lipid species were significantly altered at 24 h post-infection (hpi), 96 hpi, and 36 days post-infection (dpi), respectively. This global lipidomic profiling identified infection-specific lipid signatures for lung toxocariasis, and represented a comprehensive comparison between the lipid composition of dogs’ lungs in the presence and absence of *T. canis* infection. The potential roles of the identified lipid species in the pathogenesis of *T. canis* are discussed, which has important implications for better understanding the interaction mechanism between *T. canis* and the host lung.

## 1. Introduction

*Toxocara canis* is an important neglected zoonotic worm distributed worldwide, particularly in low-income and rural regions [1]. The definitive hosts of *T. canis* are canids [2]. *T. canis* can reside in the intestines of definitive hosts and excrete large numbers of eggs into the environment [3]. Humans can be infected with *T. canis* by the fecal–oral route [4], causing toxocariasis, such as visceral larva migrans (VLM), neurotoxocariasis (NT), and ocular toxocariasis (OT) [4,5].

In puppies and adult dogs, when *T. canis* larvae migrate to the lungs, they undergo two distinct developmental pathways, which mainly depends on the age and immune status of the host and the density of the *T. canis* larvae [6]. In puppies, when *T. canis* larvae migrate to the lungs, they can pass through the alveolar wall and continue through the bronchioles and trachea to the pharynx. Then they are swallowed by the puppy into the digestive system again, and move back to the small intestine, where larvae develop into adults and excrete eggs into the surrounding environment [7]. In adult dogs, when *T. canis* larvae migrate to the lungs, they move into different organs and muscle tissues throughout the body through the blood circulation system [7]. Therefore, the lung is a key organ that determines the migration of *T. canis*. To explore the important scientific question of why *T. canis* requires the lung migration stage during development in the definitive host, and determine the role of the lungs in the *T. canis* life cycle [8], the mechanisms of the *T. canis*–host lung interaction need to be further investigated.

The booming development of genomics, proteomics, and transcriptomics provides a unique opportunity to better understand the biology of *T. canis* [9,10]. The publication of genomic and transcriptomic data of *T. canis* marks the beginning of the era of big data in the research of *T. canis* and toxocariasis [10]. Using proteomics techniques, we can identify excretory and secretory (ES) proteins and somatic proteins of *T. canis* that contribute to immune evasion or modulation [9,11]. By analyzing the small non-protein molecules of adult *T. canis* in excretory–secretory products (ESPs), a large number of metabolites with immune modulator functions were identified, and it is noteworthy that some of the metabolites identified in *T. canis* ESPs have anti-inflammatory properties [12], providing new insights into studying *T. canis*–host interactions. Previous studies have shown that worm-derived lipids can participate in the regulation of signaling pathways during worm development and host infection [13,14].

Lipids are a class of biological organic molecules with chemical diversity that play key roles in different physiological processes [15,16]. They are important components of cell membrane and cell surface antigens, and can participate in energy metabolism [16]. Lipids are divided into eight categories according to the LIPID MAPS database (https://www.lipidmaps.org/databases/lmsd/overview (accessed on 5 November 2022)), including fatty acyls (FA), glycerolipids (GL), glycerophospholipids (GP), polyketides (PK), prenol lipids (PR), saccharolipids (SL), sphingolipids (SP), and sterol lipids (ST) [17]. Recently, untargeted metabolomics based on liquid chromatography–tandem mass spectrometry (LC-MS/MS) has provided a powerful analytical platform to comprehensively characterize lipid composition and abundance [17]. In this study, we performed a non-targeted lipidomic analysis of the lungs of Beagle dogs infected with the roundworm *T. canis*, using LC-MS/MS to identify the differential lipid species after *T. canis* infection at different stages.

## 2. Materials and Methods

### 2.1. Ethics Statement

The experimental procedures of the study were reviewed and approved by the Animal Ethics Committee of Lanzhou Veterinary Research Institute, Chinese Academy of Agricultural Sciences (Approval No. 2018-015). The Beagle dogs used in this study were handled in accordance with good animal practice as defined by the relevant Animal Ethics Procedures and Guidelines of the People’s Republic of China.

### 2.2. Animal Infection and Collection of Lung Samples

In this study, 6- to 7-week-old, specific-pathogen-free (SPF) Beagle dogs were used according to our previous description [18]. Forty-six puppies were divided into three groups according to the stage of *T. canis* migration in the canine host: 24 h group (*n* = 14, seven infected puppies vs. seven control puppies), 96 h group (*n* = 14, eight infected puppies vs. six control puppies), and 36 day group (*n* = 18, nine infected puppies vs. nine control puppies). A total of 10 litters of puppies were used, and puppies from the same litter were randomly allocated to infected groups (I) and control groups (C) to minimize background differences. Puppies in the infected group were infected orally with 1 mL of normal saline containing 300 embryonated *T. canis* eggs, and puppies in the control group were given equal amounts of normal saline only in the same manner. At 24 hpi, 96 hpi, and 36 dpi, all puppies were general anesthetized using Zoletil 50 (Virbac, France), then the KCl solution was injected into the heart of puppies for euthanasia. The lung samples from the same site in the upper lobe of the left lung of all puppies were collected and stored at −80 °C [19].

### 2.3. Sample Preparation and Lipid Extraction

First, 25 mg lung sample was placed into a 1.5 mL Eppendorf tube containing two small steel beads with 5.0 mm on a tissue grinder (50 Hz, 5 min) (JXFSTPRP, Shanghai Jingxin, China) at 4 °C. Then, 600 μL dichloromethane, 200 μL methanol, and 10 μL of SPLASH internal standards solution (330707, Avanti Polar Lipids, USA) were added to Eppendorf tube, followed by grinding using a tissue grinder (50 Hz, 5 min). Then, the ground lung sample was further processed by ultrasonic treatment (25 khz, 10 min) with a water bath at 4 °C, and was cooled at −20 °C for 1 h. Subsequently, 600 μL of supernatant was taken and lyophilized after being centrifuged at 25,000 *g* for 10 min at 4 °C, followed by reconstituting with 100 μL isopropanol, 50 μL acetonitrile, and 50 μL water. The sample was shaken for 1 min, followed by ultrasonic treatment (25 khz, 10 min), and centrifuged as above. Then, the supernatant was transferred to 1.5 mL vial. Quality control (QC) samples were prepared by pooling 20 μL of each sample extract to evaluate the stability and repeatability of LC-MS analysis.

### 2.4. The UPLC-MS/MS Analysis

In this study, the separation and detection of lipids in each lung sample and QC sample were performed using Waters 2D UPLC (Waters, USA) and a Q-Exactive high-resolution mass spectrometer (Thermo Fisher Scientific, USA). CSH C18 column (1.7 μm, 2.1 × 100 mm, Waters, USA) was used in this study. In the positive ion mode, the mobile phase consisted of solvent A (60% acetonitrile aqueous solution (ACN), 0.1% formic acid (FA) (v:v = 600:1), and 10 mM ammonium formate (AF)) and solvent B (10% ACN, 90% isopropanol, 0.1% FA (v:v:v = 100:900:1), and 10 mM AF). In the negative ion mode, the mobile phase consisted of solvent A (60% ACN and 10 mM AF) and solvent B (10% ACN, 90% isopropanol (v:v = 1:9), and 10 mM AF). The gradient elution conditions were set using mobile phase B as follows: 40–43% for 0~2 min, 43–50% for 2~2.1 min, 50–54% for 2.1~7 min, 54–70% for 7~7.1 min, 70–99% for 7.1~13 min, 99–40% for 13~13.1 min, 40% for 13.1~15 min, with a constant flow of 0.35 μL/min at 55 °C. The lipids separated by a liquid phase were injected into the Q-Exactive mass spectrometer (Thermo Fisher Scientific, USA) to obtain MS1 and MS2 data in the range of 200–2000 m/z. In the MS1 analysis, MS scan settings were resolution 70,000, maximum injection time (MIT) 100 ms, automatic gain control (AGC) 3E6. For subsequent MS2 analysis (resolution, 17,500; MIT, 50 ms; AGC, 1E5), the top three ions were selected according to the precursor ion intensity. Collision energies (stepped and normalized collisional energy) were set as 15, 30, and 45 eV.

### 2.5. Data Analysis

In this study, LC-MS/MS technology was used for non-targeted lipidomics analyses, and a high-resolution mass spectrometer Q Exactive (Thermo Fisher Scientific, Waltham, MA, USA) was used to collect data in positive and negative ion modes to increase lipid detection coverage, respectively. LC-MS/MS data analysis was performed using LipidSearch v.4.1, including intelligent peak extraction, lipid identification and peak alignment, and subsequent data analysis. The following parameters were used for lipid identification and peak extraction: product was selected as the identification type, the quality deviation of precursor ions and product ions in the library was 5 ppm, and the response threshold was set as the relative response deviation of product ion 5.0%; the quantitative parameter was set to calculate the peak areas of all identified lipids, and the mass deviation of peak extraction was set to 5 ppm; the filter is set as the top-rank, all-isomer peak, FA priority, M-score was set as 5.0, c-score was set as 2.0, and the identification level was selected as “A”, “B”, “C”, and “D”. All lipid categories were selected for identification, and adduct forms of positive ion mode were [M+H]^+^, [M+NH4]^+^, [M+Na]^+^, and of negative ion mode were [M-H]^−^, [M-2H]^−^, [MHCOO]^−^.

Statistical analysis was performed through the metabolomics metaX R package (1.4.2) (https://www.bioconductor.org/packages/3.3/bioc/html/metaX.html, accessed on 5 November 2022) [20], including deletion of lipid species missing more than 50% in QC samples and more than 80% in experimental samples, normalization of the data using the probability quotient normalization (PQN) method [21], and deletion of the lipid species with a relative peak area CV (coefficient of variation) > 30% in all QC samples. Data quality was evaluated by detecting the reproducibility of QC samples, including chromatograms overlap of QC samples, principal component analysis (PCA), peak number, and peak response intensity difference. Quantitative analysis of lipid subclasses was performed for relative content in the infected and control groups. The PCA and partial least squares discriminant analysis (PLS-DA) were used for the multivariate statistical analysis. Fold change (FC) and *t*-test analysis were used for univariate analysis. Screening conditions for differential lipid species were VIP ≥ 1, FC ≥ 1.2 or ≤0.8333, and *p* value < 0.05. Additionally, the differential lipid species were clustered using the hierarchical clustering method, and the Euclidian distance was used for distance calculation and Z-score (zero-mean normalization) for data normalization. 

## 3. Results

### 3.1. The Attributes of Lipid Profiles in Lung

The base peak chromatograms (BPC) of all QC samples have good overlap, little fluctuation of retention time, and peak response intensity, showing that the signal output of QC samples were stable during the entire detection and analysis process (Supplementary Figure S1). According to the relative standard deviation (RSD) ≤ 30% in all QC samples, a total of 1197 lipid species were identified; among these, 574 and 623 were identified in the positive ion mode (ESI+) and negative ion mode (ESI−), and the ratio of the effective lipid species number was 92%, which was calculated by the number of lipid species with CVs of relative peak area less than or equal to 30% of the number of all detected compounds in the QC samples (Supplementary Tables S1 and S2). The ratio ≥ 60% indicates that the data quality is qualified. All QC samples were clustered closely in PCA analysis, and the RSD value of 87.5% for identified lipids was less than 20% (Supplementary Figure S2).

**Figure 1:**
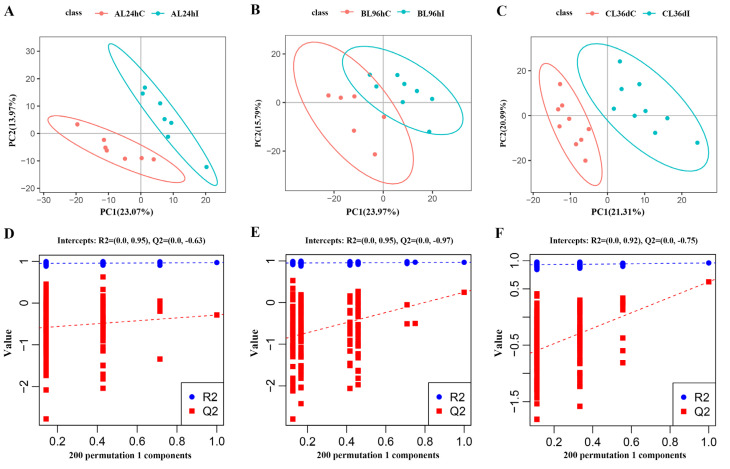
The partial least squares discriminant analysis (PLS-DA) model score map (**A**–**C**), and the response sequencing verification diagram of the PLS-DA model (**D**–**F**) between the infected group (I) and control group (C) at 24 hpi, 96 hpi, and 36 dpi, respectively.

The PCA analysis showed that the infected group and control group cannot be well separated at 24 hpi, 96 hpi, or 36 dpi (Supplementary Figure S3), whereas the infected group and control group can be readily distinguished by PLS-DA analysis, indicating that the infection model has been successfully established (Figure 1A–C). Permutation tests were performed with 200 responses to judge the quality of the model, which showed that the values of R^2^ and Q^2^ were 0.89 and −0.62, 0.91 and −0.48, and 0.91 and −0.71 at 24 hpi, 96 hpi, and 36 dpi, respectively (Figure 1D–F).

### 3.2. The Lung Lipidomic Changes

**Figure 2:**
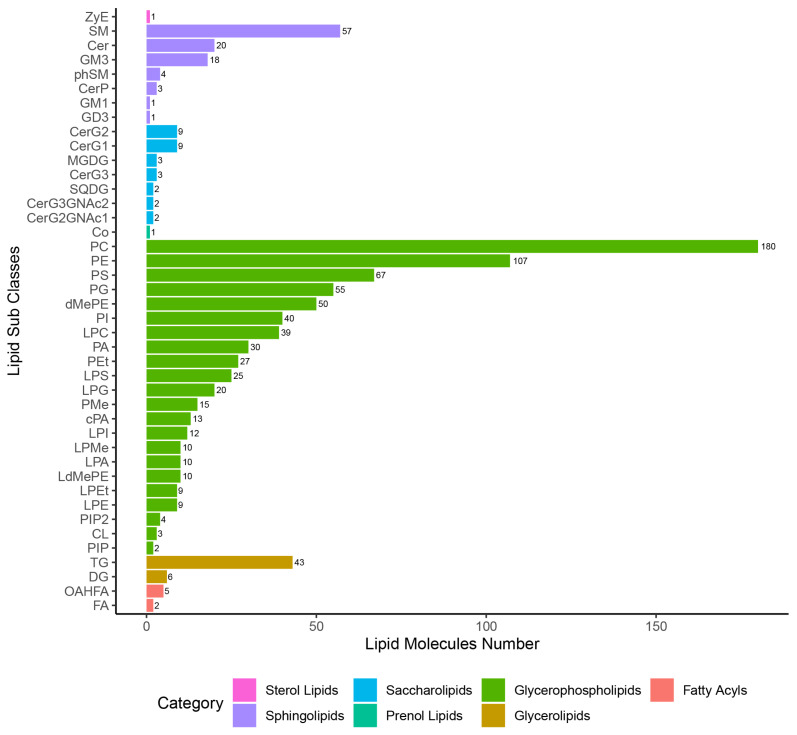
Statistical chart of lipid subclasses and corresponding number of lipid species.

In this study, seven categories of lipids were identified, namely ST, SL, GP, FA, SP, PR, and GL in QC samples and each of the experimental samples. After eliminating the 268 lipid species that were repeated lipid identification results (Supplementary Table S3), a total of 929 lipid species were left for subsequent allocation into lipid subclasses and corresponding number of lipid species analysis (Figure 2). Among these, GP was the most abundant subclass with 737 species; ST and PR were the least abundant subclass with only one species. A total of 42 lipid subclasses were identified, as well as three, five, and 14 lipid subclasses that were altered at 24 hpi, 96 hpi, and 36 dpi, respectively, which belong to the categories GL, GP, or SP, respectively (Supplementary Figure S4). In total, 40% (2/5) of lipid subclasses in the GL category were significantly altered at 24 hpi or 96 dpi, including diglyceride (DG) and triglyceride (TG). Moreover, 50% (4/8) lipid subclasses in the SP category were significantly altered at 36 dpi, including ceramides (Cer), sphingomyelin (SM), and ceramide phosphate (CerP).

**Figure 3:**
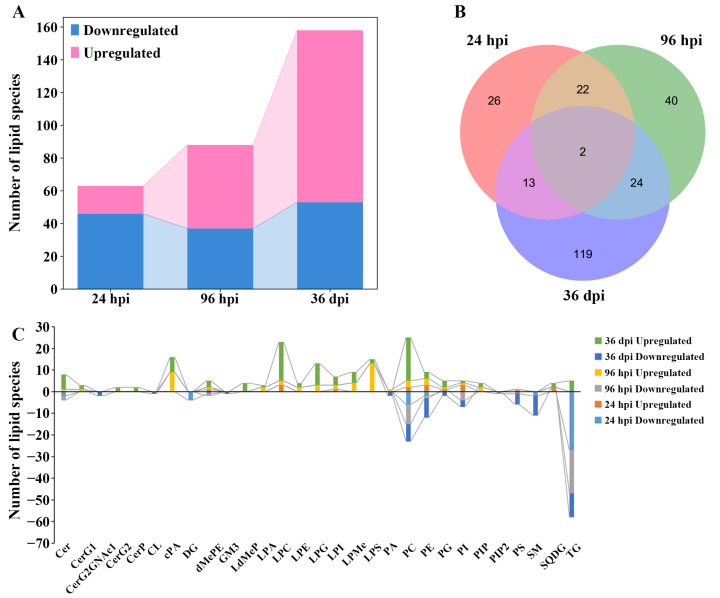
The comparisons of lipids with differential abundance in the lungs of Beagle dogs infected with 300 embryonated *Toxocara canis* eggs at 24 hpi, 96 hpi, and 36 dpi. (**A**): The number of lipid species with differential abundance are shown in the stacked bar chart at the three infection stages. (**B**): The common and unique differential lipid species visualized with a Venn diagram among the three infection stages. (**C**): The number of lipid species with differential abundance in each lipid subclass at 24 hpi, 96 hpi, and 36 dpi, respectively.

Overall, 63, 88, and 157 differential lipids were identified at 24 hpi, 96 hpi, and 36 dpi, respectively (Figure 3A, Supplementary Figure S5, and Table S4). At 24 hpi, 46 differential lipids were downregulated and 17 differential lipids were upregulated, which belong to 14 lipid subclasses, such as TG (18:2/18:2/18:2), lysophosphatidylcholine (22:5, LPC (22:5)), and DG (16:0/18:2). At 96 hpi, 37 differential lipids were downregulated and 51 differential lipids were upregulated, which belong to 18 lipid subclasses, such as TG (16:0/18:1/18:1), lysophosphatidylserine (20:0, LPS (20:0)), and PC (18:0/22:6). At 36 dpi, 53 differential lipids were downregulated and 105 differential lipids were upregulated, which belong to 27 lipid subclasses, such as TG (16:0/16:0/16:1), lysophosphatidylglycerol (22:5, LPG (22:5)), and phosphatidylserine (20:2/20:2, PS (20:2/20:2)). Furthermore, two differential lipids were commonly identified between the three infection stages, namely TG (16:1/18:1/18:2) and TG (16:0/18:1/19:0) (Figure 3B). The Venn diagrams show that 22 common differential lipid species were identified at 24 hpi and 96 hpi, and all differential lipids were downregulated in these two infection stages; 24 common differential lipid species were identified at 96 hpi and 36 dpi, with two downregulated differential lipids and 22 upregulated differential lipids in these two infection stages.

The number of differential lipids was calculated in each lipid subclass, which showed that some differential lipids of diglyceride (DG, four lipid species) and phosphatidylinositol 4,5-bisphosphate (PIP2, one lipid species) were identified only at 24 hpi; and some differential lipids of lysodimethylphosphatidylethanolamine (LdMePE, four lipid species), diglycosylceramide (CerG2, two lipid species), and ceramide phosphate (CerP, two lipid species) were identified only at 36 dpi. Furthermore, some differential lipids of the monoglucosylceramide (CerG1, one and two lipid species), cyclic phosphatidic acid (cPA, nine and seven lipid species), and lysophosphatidylglycerol (LPG, three and 10 lipid species) were identified at 96 hpi and 36 dpi; and differentially abundant lipid ceramide species (Cer, two, three, and seven lipid species), phosphatidylcholine (PC, eight, 12, and 28 lipid species), and triglyceride (TG, 27, 20, and 16 lipid species) were identified at 24 hpi, 96 hpi, and 36 dpi, respectively (Figure 3C). In addition, the abundance of differential lipids was clustered at different infection stages, showing that the infected group and control group can be separated by hierarchical cluster analysis at each infection stage (Supplementary Figure S6). Therefore, lipidomics is a reliable approach for identifying lipids with different abundances.

## 4. Discussion

Lipidomics is a powerful strategy to understand the structure and function of lipids, by which the composition (categories, subclass, and molecular species) and content changes of lipids in the body can be explored, and the mechanism of lipid metabolism regulation in various life phenomena can be revealed [22]. In this study, the LC-MS/MS technique was used for the lipidomics analysis, and 63, 88, and 157 differential lipids were identified at 24 hpi, 96 hpi, and 36 dpi, respectively. The number of downregulated differential lipids was greater than that of the upregulated differential lipids at 24 hpi; however, the number of upregulated differential lipids was greater than that of the downregulated differential lipids at 96 hpi and 36 dpi. A previous study showed that *T. canis* larvae were found in the lungs of all infected puppies at 96 hpi, but without obvious clinical symptoms [19]. Eosinophilia was found in puppies infected with *T. canis* at 24 hpi, 96 hpi, and 36 dpi [23], which is a common feature of host after helminth infection [24]. Furthermore, anti-*T. canis* IgG antibodies were detected at 36 dpi in infected puppies [19]. In this study, 14 lipid subclasses were significantly altered at 36 dpi, which was far more than that at 24 hpi or 96 hpi.

At the early stages of the infection, three altered lipid subclasses (TG, sulfoquinovosyldiacylglycerol (SQDG), and DG) and 63 differential lipids were identified at 24 hpi. Five altered lipid subclasses (lysophosphatidylserine (LPS), TG, phosphatidylinositol phosphate (PIP), cyclic phosphatidic acid (cPA), and phosphatidylcholine (PC)) and 88 differential lipids were identified at 96 hpi. *T. canis* larvae were found in the lungs of all infected puppies at 96 hpi [19]. Previous studies have shown that the invasion of helminths is able to alter the lipid metabolism in the host and to affect the lipid metabolism of immune cells [25,26].

The downregulation of DG 1.65 times, including four downregulated DG lipid species at 24 hpi, while without significant changes in DG at 96 hpi and 36 dpi, suggests that these lipids may play a role at the early stage of *T. canis* infection. The abundance of TG was the most significant alteration, which was downregulated 1.57 times and 1.58 times, including 27 downregulated and 20 downregulated TG lipid species at 24 hpi and 96 hpi, respectively. TG is hydrolyzed to provide FA for many cellular processes, such as lipid synthesis and energy production [27]. Additionally, lysophosphatidylserine (LPS) is an amphipathic lysophospholipid, which mediates the inflammatory response [28]. By studying the role of LPS in airway epithelial cells that were involved in lung homeostasis, it was found that LPS has a potential receptor-independent role during inflammation [28], which provides new insights for subsequent studies. At 96 hpi, LPS was upregulated 1.33 times, including 12 upregulated LPS lipid species. LPS is a deacylated form of phosphatidylserine, and is known as an important class of a bioactive glycophospholipid mediator, which has various immunomodulatory functions, primarily on immune cells, especially mast cells [28]. Studies have shown that elevated LPS concentrations can cause an inflammatory response [29,30]. LPS from schistosomes was identified as a TLR2-activating molecule, and acts on dendritic cells to induce regulatory T cells [31]. Studies have shown that LPS displays immunoregulatory activities by GPCR, as well as differentiates monocytes to produce mature dendritic cells, and stimulates the production of IL-2 and IFN-γ by allogeneic T lymphocytes [32,33]. The role of LPS in the immune response against the worm can be summarized as follows: (1) the LPS is able to promote the membrane fusion with the host cell, so that the worm can obtain the host membrane components; (2) LPS may alter the immune recognition of worm antigens [34]. These properties may be the main mechanism by which the worm escapes from the host. We speculate that LPS may play a role in attenuating host immunity and facilitating better worm survival. However, further studies are required regarding the effect of LPS on *T. canis*. 

PC is one of the most abundant phospholipids found in all cell membranes [35], and accounts for nearly 50% of total membrane phospholipids [36,37]. The PC was downregulated 1.09 times and 1.06 times, including nine downregulated and eight downregulated PC lipid species at 96 hpi and 36 dpi, respectively. PC is a precursor to produce glycoconjugates secreted by nematodes to avoid host immune responses [38]. Nematodes evade the host immune response by modifying glycoproteins and glycolipids with PC [39]. A previous study has shown that multiple pathways of PC biosynthesis may be potential inhibitor targets in metabolic variations between nematodes and *Plasmodium* [38]. Therefore, identification of different biochemical targets between *T. canis* and the host is crucial for further investigation. Moreover, PIP is a phospholipid, and thus is an important component of cell membranes [40]. At 96 hpi, PIP was upregulated 2.05 times, including two upregulated lipid species of PIP. PIP can be involved in the regulation of various cellular functions [41]. Studies have shown that PIP signaling pathways play an important role in cellular processes, such as membrane trafficking, cell signal transduction, and reproductive systems [42,43]. Previous studies have found that the phosphorylated PIP form in *C. elegans* has multiple functions, which play important roles in diapause [44] and thermotactic behavior [45]. However, there are no relevant studies of the effect of PIP on *T. canis*, thus further research is needed.

At 36 dpi, 14 lipid subclasses were significantly altered, such as ceramides (Cer), ceramide phosphate (CerP), phosphatidylserine (PS), dimethylphosphatidylethanolamine (dMePE), lysophosphatidylglycerol (LPG), lysophosphatidylinositol (LPI), and cardiolipin (CL). Cer is the precursor of the entire glycosphingolipid family [46]. It has been found that Cer can act as a second messenger in the transduction of cell signaling [47]. The Cer subclass was upregulated 1.23 times, including seven upregulated lipid species at 36 dpi. Cer is a key lipid subclass in signaling cascades and energetic–metabolic pathways, modulating critical physiological functions in cells [48]. Cer plays an important role in the alteration of membrane structures; for example, these lipids can trigger the formation of channel-like structures in the outer mitochondrial membrane (OMM), which can alter permeability and ion concentration [48], suggesting that these Cer lipids may be functionally important for the migration of *T. canis*. Sphingomyelins (SMs) are an important class of lipids that are widely found in different organisms [49]. SM is enriched in the central nervous system, where it is a component of the myelin sheath [50]. SM is the most abundant sphingolipid (SP) in cells [51], and has the ability of binding cholesterol [52]. The hydrolysis of SM can increase the concentration of Cer, by which Cer is involved in cell proliferation, growth, and apoptosis [51]. At 36 dpi, SM was downregulated 1.14 times, including nine downregulated lipid species, such as SM (d36:4), SM (d38:4), and SM (d44:5). *T. canis* reinfestations may occur, evolving into neurotoxocariasis, the diagnosis of which remains difficult because it generally requires central nervous system (CNS) examination [53]. On this view, the changes in CNS tissue lipid levels strongly indicate the pathological changes occurring in CNS tissue following cell damage, and may serve as novel biomarkers in the diagnosis of neurotoxocariasis. At the later stage of *T. canis* infection, cardiolipin (CL), sphingomyelin (phSM), and dimethylphosphatidylethanolamine (dMePE) were downregulated 1.21, 1.13, and 1.08 times, respectively; however, the function of these metabolites remains unclear in worm infection, especially in *T. canis* infection.

## 5. Conclusions

In this study, global lipidomics analyses were performed by LC-MS/MS to compare the lipid composition and content changes in Beagle dog lungs at different stages of *T. canis* infection. Lung lipid profiles were different between the infected dogs and uninfected control dogs at the different infection stages. Further exploration into the function of these differential lipids, such as TG, LPS, and Cer, will contribute to elucidating the mechanisms underlying *T. canis* pathogenesis in the lung of the canine host.

## Data Availability

The datasets supporting the findings of this article are included within the article. The metabolomic data are available in the MetaboLights database under Accession No. MTBLS5583 (http://www.ebi.ac.uk/metabolights/MTBLS5583, accessed on 5 November 2022).

## Supplementary Materials

The following are available online at https://www.mdpi.com/article/10.3390/ani12223080/s1. Table S1. The effective lipids and its expression levels after normalization in each sample at 24 hpi, 96 hpi, and 36 dpi in positive ion mode (ESI+) and negative ion mode (ESI−). Table S2. All of the detected lipids at 24 hpi, 96 hpi, and 36 dpi. Table S3. After eliminating the equivocal lipids species, the effective lipid species and their expression levels after normalization in each sample at 24 hpi, 96 hpi, and 36 dpi in positive ion mode (ESI+) and negative ion mode (ESI−). Table S4. The differential abundance of lipids at 24 hpi, 96 hpi, and 36 dpi. Figure S1. The base peak chromatograms (BPC) overlapping spectrum of QC samples in positive mode (A) and negative mode (B). Figure S2. The coefficient of variation (CV) distribution of lipid species and principal component analysis (PCA) of QC samples. A: In the figure, the two lines perpendicular to the *X*-axis are 20% and 30% CV reference lines, respectively, and the lines parallel to the *X*-axis are 60% reference lines. B: The PCA score scatter plots of lipids in QC of infected (I) and control (C) samples. Figure S3. The scatter plot of the principal component analysis (PCA). A–C represent PCA score plots of the infected groups (I) and control groups (C) at 24 hpi, 96 hpi, and 36 dpi, respectively. Figure S4. The changes of lipid subclasses between the infected group (I) and control group (C) by Student’s *t*-test using SPSS 19 at 24 hpi, 96 hpi, and 36 dpi, respectively. * *p* < 0.05, ** *p* < 0.01, *** *p* < 0.001. Figure S5. Volcano plots showing the differentially expressed lipids at 24 hpi, 96 hpi, and 36 dpi, respectively. The blue color of the dots represents significantly downregulated lipids, and the red color of the dots represents significantly upregulated lipids. Figure S6. Heatmaps and hierarchical clustering of the lipid species with differential abundance between the infected groups (I) and control groups (C) at 24 hpi (A), 96 hpi (B), and 36 dpi (C). Each row represents a differential lipid, and each column represents a sample.
