# Supplementary material for: Lung Lipidomic Alterations in Beagle Dogs Infected with Toxocara canis
Source: Animals (Basel). 2022 Nov 9;12(22):3080. doi: 10.3390/ani12223080 (PMC9686702; doi:10.3390/ani12223080)
Supplement: Supplementary file 1 [file animals-12-03080-s001.zip › Supplementary Figures.pdf]

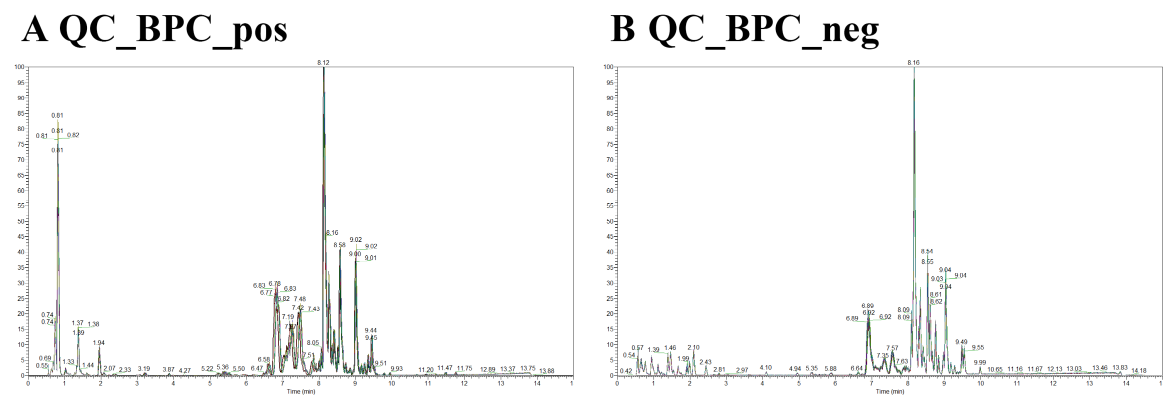

Figure S1. The base peak chromatograms (BPC) overlapping spectrum of QC samples in positive mode (**A**) and negative mode (**B**).

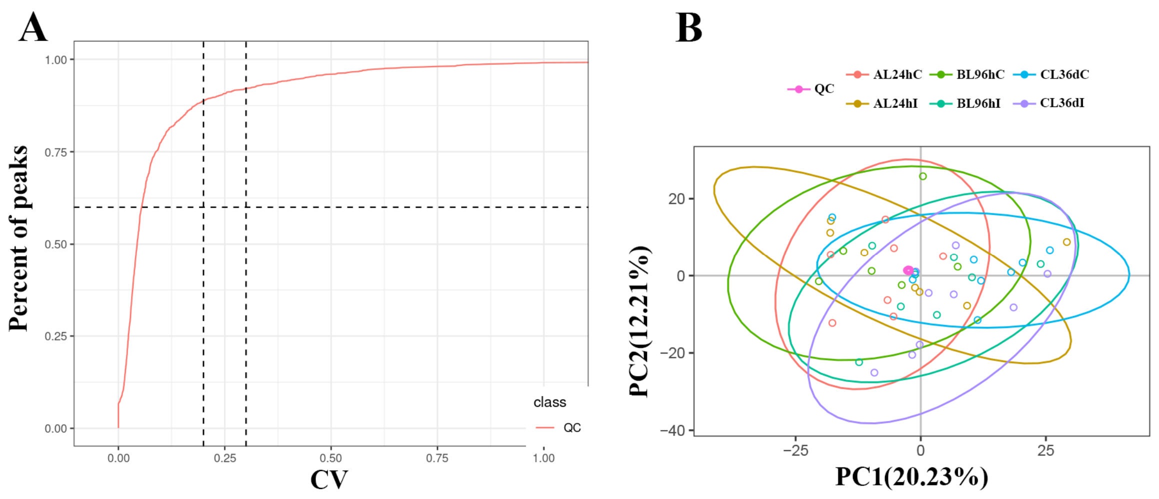

Figure S2. The coefficient of variation (CV) distribution of lipid species and principal component analysis (PCA) of QC samples. **A**: In the figure, the two lines perpendicular to the *X*-axis are 20% and 30% CV reference lines, respectively, and the lines parallel to the *X*-axis are 60% reference lines. **B**: The PCA score scatter plots of lipids in QC of infected (I) and control (C) samples.

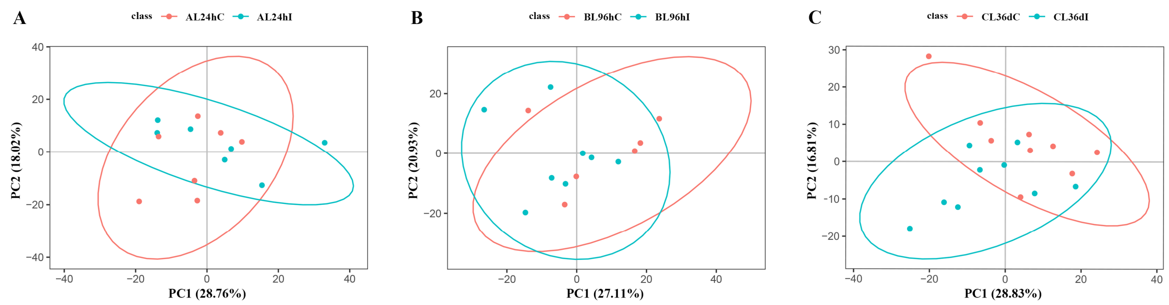

Figure S3. The scatter plot of the principal component analysis (PCA). **A–C** represent PCA score plots of the infected groups (I) and control groups (C) at 24 hpi, 96 hpi, and 36 dpi, respectively.

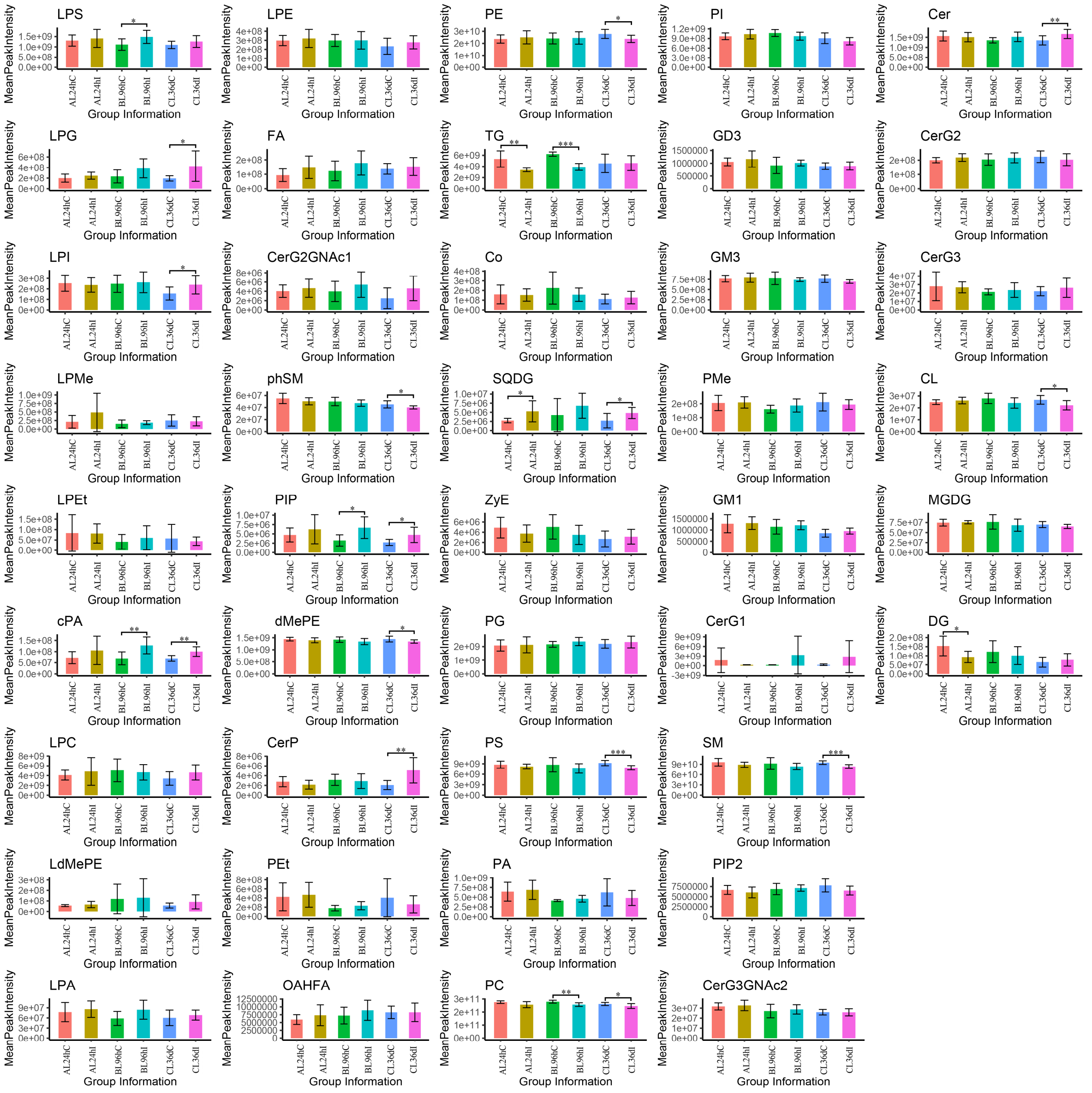

Figure S4. The changes of lipid subclasses between the infected group (I) and control group (C) by Student's *t*-test using SPSS 19 at 24 hpi, 96 hpi, and 36 dpi, respectively. * $p < 0.05$, ** $p < 0.01$, *** $p < 0.001$.

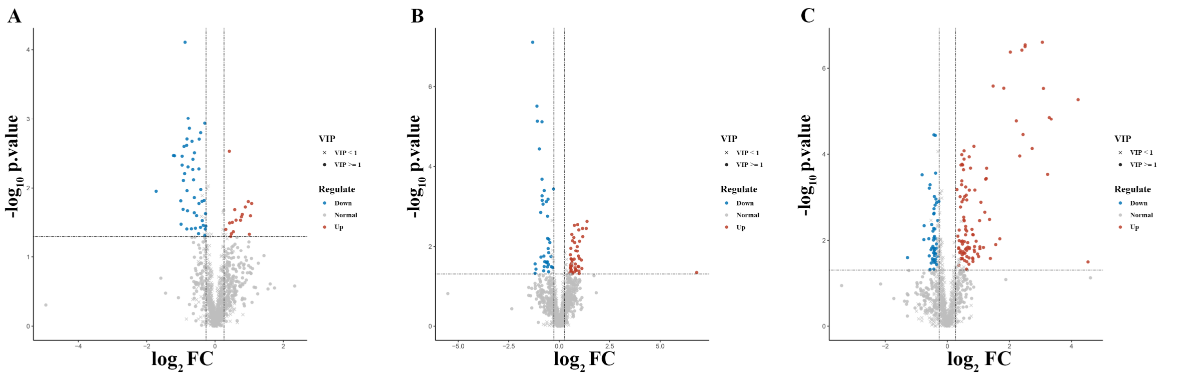

Figure S5. Volcano plots showing the differentially expressed lipids at 24 hpi, 96 hpi, and 36 dpi, respectively. The blue color of the dots represents significantly downregulated lipids, and the red color of the dots represents significantly upregulated lipids.

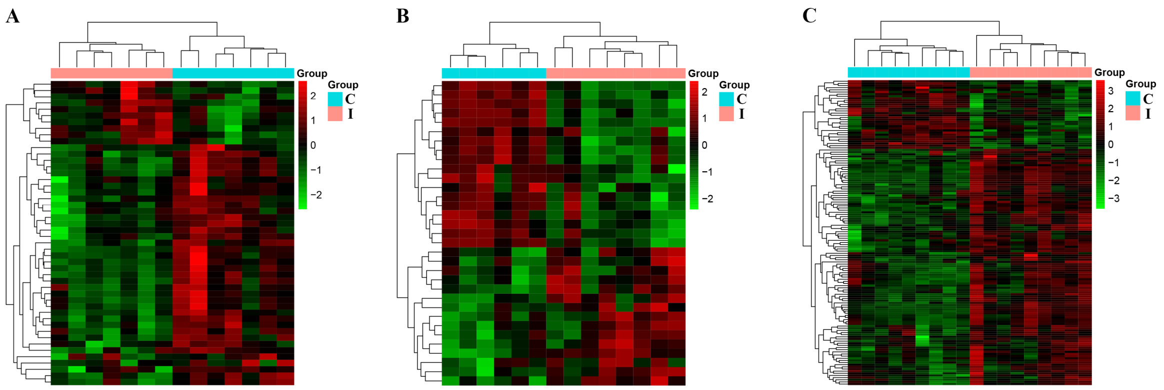

Figure S6. Heatmaps and hierarchical clustering of the lipid species with differential abundance between the infected groups (I) and control groups (C) at 24 hpi (**A**), 96 hpi (**B**), and 36 dpi (**C**). Each row represents a differential lipid, and each column represents a sample.
